# Modulatory Effect of Gonadotropins on Rats’ Ovaries after Nandrolone Decanoate Administration: A Stereological Study

**Published:** 2014-01

**Authors:** Hossein Bordbar, Fakhroddin Mesbah, Tahereh Talaei, Farzaneh Dehghani, Hossein Mirkhani

**Affiliations:** 1 Stereology and Histomorphometry Research Center, School of Medicine, Shiraz University of Medical Sciences, Shiraz, Iran;; 2 Department of Anatomical Sciences, School of Medicine, Shiraz University of Medical Sciences, Shiraz, Iran;; 3 Department of Pharmacology, School of Medicine, Shiraz University of Medical Sciences, Shiraz, Iran

**Keywords:** Nandrolone decanoate, Gonadotropin, Ovary

## Abstract

**Background:** Nandrolone decanoate (ND) is an anabolic androgenic steroid (AAS) which influences the ovarian structure and function. We assessed the effects of ND on the ovarian volume, number of primordial follicles, and level of hormones and also evaluated the modulatory effects of gonadotropins on the histopathological changes imposed by the administration of ND.

**Methods:** Six groups of Sprague-Dawley adult female rats (n=30) were used. The experimental rats were injected intraperitoneally with 3 and 10 mg/kg ND with or without human menopausal gonadotropin (hMG), 10 IU weekly for one month. The vehicle and control rats were administered olive oil and saline, respectively, for the same period of time. The ovarian volume and number of primordial follicles were estimated by stereological methods.

**Results: **The results showed a decrease in the ovarian volume, number of primordial follicles, and level of gonadotropins in the ND-treated animals compared with the vehicle groups. In the rats treated with 3 mg/kg of ND with hMG, an increase in the ovarian volume and number of primordial follicles was shown as compared to the rats treated with the same dose of ND without hMG.

**Conclusion: **ND exerted detrimental effects on the dimensions of the ovary, number of follicles, and level of sex hormones. However, hMG, prevented the harmful effects of ND (at least in a low dose) on the ovarian follicles.

## Introduction

Pharmaceutical usage of anabolic androgenic steroids (AASs) has led to an increase in the incidence of infertility among young couples. These steroids impose some reproductive disorders through counteracting gonadotropins. Although athletes are the main consumers of AASs to increase their muscle mass, non-athletes undergoing incorrect fitness or bodybuilding courses also take these drugs to reduce their body fat. Moreover, AASs are taken to improve self-esteem, cross-gender competition, and self-protection in women.^1^^,^^2^

Numerous studies have been performed on the effects of AASs on ovarian follicles. It has been shown that AASs induce apoptosis in the follicular cells of rats’ antral follicles.^3^^,^^4^ They also influence the morphology of the uterus and ovaries, causing histopathological changes, including epithelial vacuolization and endometrial stromal fibrosis, and reducing the reproductive capacity in rats.^3^^,^^5^ AASs can also disrupt the hypothalamic-hypophyseal-gonadal axis and decrease the serum level of gonadotropins.^6^ A stereological study showed that follicle-stimulating hormone (FSH) increases the number of the ovarian follicles in rats due to a synergy it creates with gonadotropins.^7^

Nandrolone decanoate (ND) is an AAS which is widely used nowadays. These drugs make it act as potential male sex hormones. ND reduces FSH and luteinizing hormone (LH) secretion through a negative feedback mechanism and subsequently leads to menstrual and follicular disorders.^2^^,^^8^

Almost all the studies conducted on the effects of ND on the histomorphology of the ovary and uterine tissues, have unanimously reported a significant decrease in the antral follicle count and changes in the uterine tissue morphology.^9^^,^^10^ Recent studies have shown reduced number of antral follicles on rat ovarian tissue and increased epithelial as well as the endometrial stromal thickness and estral acyclicity.^5^^,^^8^

Therefore, as was shown in the above mentioned studies, structural and functional modifications in the ovarian follicles might increase the infertility rate. The objectives of the present study were to determine the effects of ND on the volume of the ovarian cortex and medulla and the number of primordial follicles and to assess the modulatory effects of gonadotropins on the histopathological changes imposed by ND.

## Materials and Methods

Thirty female Sprague-Dawley rats weighing 180-210g were selected randomly and kept under constant conditions of light (12 h light/dark cycles) and temperature (21-24ºC). The experimental protocols and animal handling procedures were reviewed and approved by the Animal Ethics Committee of Shiraz University of Medical Sciences.

*Experimental Design*

The animals were randomly divided into six groups (n=5). The first group received 3 mg/kg (low dose) intraperitoneal (IP) administration of ND (GMBH Hamburg Germany) weekly for four weeks. The second group received 10 mg/kg (high dose) for the same period of time. The third group received 0.1 ml IP administration of olive oil as vehicle (Darou Pakhsh Tehran Iran) for the same period of time (vehicle 1). The fourth and fifth groups received 10 IU of human menopausal gonadotropin (hMG) (Institut Biochimique SA-CH, Tehran, Iran) IP administration concurrent with the 3 mg/kg ND (low-dose hMG) and 10 mg/kg ND (high-dose hMG) for four weeks. The sixth group received olive oil and saline in the same volume IP administration (vehicle 2). Vaginal smear was performed before every single injection to make sure that the animals were at the estrous phase of the estrous cycle. At the end of the experiment, blood samples were taken from the rats’ tails (1 mL) in order to determine the levels of FSH, LH, estrogen, and progesterone. Then, the rats were scarified using anesthetic diethyl ether. The ovaries of each animal were removed and fixed in 10% formalin fixative for 24 h before they were dehydrated and embedded in paraffin.

*Stereological Methods*

The orientated method was used to obtain isotropic uniform random (IUR) sections.^11^ The paraffinized ovaries were sectioned serially in 5 µm thickness (H&E staining) for volume estimation and 25 µm thickness (Feulgen staining) for number**.**

*Estimating the Number of Primordial Follicle*

**Figure 1:**
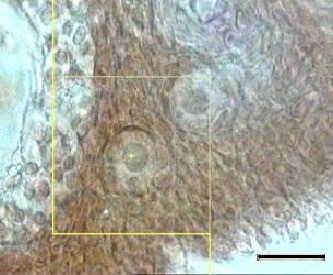
Estimation of the total number of the primordial follicles in the rats, using the optical disector method. An unbiased counting frame is superimposed on the images. Any oocyte nuclei that come into the maximal focus within the traveling optical section are counted. The nucleoli profiles of the oocytes are counted in the next 10 µm of the 25 µm section. Scale bar=10 µm

Morphological classification of the follicles in the rats was determined as primordial if a follicle contained an oocyte surrounded by a partial or complete layer of squamous granulose cells. The number of primordial follicles was determined using an optical disector design applied to 25 µm thick sections. This method is based on the direct counting of the particles (in this study, the oocyte nucleoli) in the original structure. The total numbers of the primordial follicles was estimated by stereological software. The unbiased counting frame was superimposed on the images that were viewed on the monitor (figure 1). An average of 80-100 microscopic fields were selected in each ovary via a systematic sample. The position of the first area was selected randomly outside the sections and the other areas were selected by moving the microscope stage in an equal interval along the x- and y- directions using a stage micrometer. A high numerical aperture (×100 magnification)-(NA=1.4) oil- immersion lens was used. The final magnification was ×60 using a microcator (Heidenhain MT-12 Germany), which measures the z-axis traveling. Any nucleolus in focus at the starting 5µm plane was excluded. Any nucleolus which came into maximal focus within the next traveling 5µm optical section (height or disector) was selected if it lay in the counting frame or touched the inclusion border and did not touch the exclusion borders or the frame. The numerical density of the primordial follicles was estimated using the following formula:^11^

$${\text{N}}_{\text{V}}=\frac{\sum \text{Q}}{\sum \text{p}\times a\left(\text{f}\right)\times \text{h}}$$

where “∑Q” is the total number of the counted cells “h” is the tissue thickness (10 µm) considered for counting “a/f” is the frame area in the true tissue scale and “∑p” is the total number of the points superimposed on the selected fields. The result of the equation was then multiplied by the total volume of the ovary to obtain the total number of the primordial follicles.

*Estimating the Volume of Ovarian Cortex and Medulla*

**Figure 2:**
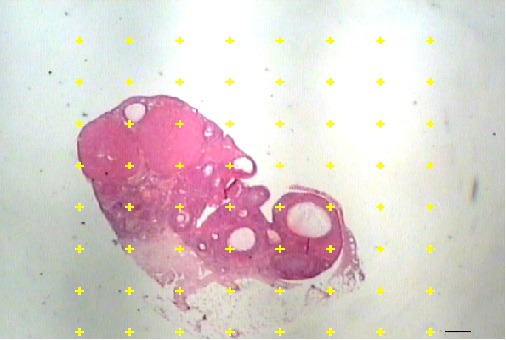
Estimation of the volume density of the ovarian cortex and medulla in the rats, using the Cavalieri principle. The total number of the points hitting each component is divided by the total number of the points hitting the reference space. Scale bar=1 µm

This estimation was performed by the Cavalieri method. After staining with H&E, 10-12 sections were selected in a systematic random manner and examined using a video-microscope at ×1 magnification. The ovarian volume and the volumes of the cortex and medulla were obtained by point counting method (figure 2) and the following formula:^11^

$$\text{V}=\sum \text{p}\times \left(\frac{\text{a}}{\text{p}}\right)\times \text{t}$$

where “∑p” is the total number of points hitting the sections; “a/p” is the area per point; and “t” is the distance between the sampled sections. Additionally, “a/p” is calculated by the following formula:

$$\left(\frac{\text{a}}{\text{p}}\right)=\frac{\left(\Delta \text{x}\times \Delta \text{y}\right)}{{\text{m}}^{\text{2}}}$$

where “Δx” and” Δy” are the distance between the two adjacent points on the grid in the x-axis or the y-axis, respectively. Moreover, “m” is the final linear magnification of the microscopic images. The total number of the follicles was estimated using the following formula:^11^

$$\text{N}={\text{N}}_{\text{v}}\times \text{V}$$

where “NV” is the number density of primordial follicles; and “V” is the ovarian volume.

*Blood Sampling and Hormone Assay*

The blood samples, which were collected from the rat’s tails both before and after the treatment were centrifuged at 4°C for 10 min at 250 g. The serums were stored at -20°C until the biochemical analysis. The concentrations of the serum hormones (FSH LH estrogen and progesterone) were determined using the RAT FSH/LH (Shibayagi Co Tokyo Japan) and Estrogen/Progesterone (Cusabio, Co, China) ELISA Kit. The samples were incubated in monoclonal anti-LH/FSH/estrogen/progesterone antibody and measured spectrophotometrically at 450 nm. Intra assay and inter assay mean were less than 5%.

*Statistical Analysis*

The data were analyzed through the Kruskal-Wallis non-parametric test. A P value less than 0.05 was considered statistically significant. The statistical analyses were done using the SPSS statistical software (version 15).

## Results

The descriptive statistics of the cortical, medullary, and total volumes of the rats’ ovaries as well as the number density and total number of the primordial follicles are depicted in table 1. The results showed that the low and high doses of ND decreased the cortical and total volumes of the ovaries significantly (P=0), while the ovarian cortex and the total volume of the ovary were increased significantly in the rats that received hMG in combination with ND (P=0). ND did not have any effect on the medullary volume, but the volume of the cortex and medulla in the low-dose hMG-treated animals increased compared to the high-dose hMG-treated group; the difference, however, did not constitute statistical significance.

**Table 1:** Mean value and standard deviation (SD) of the cortical, medullary, and total ovary volumes and the mean value and SD of the number density of the ovarian primordial follicles treated with a low-dose of ND and high-dose of ND, alone or with hMG and vehicle groups

	** OCV (mm^3^) ** **mean±SD**	** OMV (mm^3^) ** **mean±SD**	** OV (mm^3^) ** **mean±SD**	**NDPF ** **mean±SD**	**TNP ** **mean±SD**
LDN	7.6460±1.19828^a^	9.4200±0.56476	16.6740±1.27720^a^	3903.2±148.22854^a^	39918±1461.85584^a^
HDN	6.8680±0.67585^a^	8.8280±0.46122	15.3080±0.54353^a,c^	3312±144.23418^a,c^	36165.6±594.76323
Vehicle 1	11.4080±0.95224	9.9260±0.72923	21.1400±0.78514	4863.6±89.71232	50982.6±1193.75136
LDN+hMG	12.7540±0.64489^b,c,d^	9.8520±0.94036	21.2140±0.67095^b,c,d^	5054.6±106.34989^b,c,e^	52061.4±1414.09558^b,c,e^
HDN+hMG	10.6960±1.26037^c,d^	8.3160±1.07658^c,d^	19.4180±1.57630^c,d^	4814.2±62.34340^c,d^	49452.4±12382.74372^c,d^
Vehicle 2	11.6800±0.87204	9.9420±0.58277	20.0240±0.91500	4868.4±30.94027	50495.2±732.32759

a: Compared to vehicle 1 (P=0); b: Compared to vehicle 2 (P=0); c: Compared to LDN group (P=0); d: Compared to HDN group (P=0); e: Compared to HDN+hMG group (P=0); hMG: Human menopausal gonadotropin; LDN: Low-dose Nandrolone; HDN: High-dose Nandrolone; Vehicle 1 (olive oil) vehicle 2 (olive oil+saline); OCV: Ovarian cortical volume; OMV: Ovarian medullary volume; OV: Ovarian volume; NDPF: Number density of the primordial follicle; TNPF: Total number of the primordial follicle

The total number and number density of the primordial follicles were decreased significantly by ND (P=0), while they were increased in the animals that received a combination of low-dose ND and hMG. In addition, in the high-dose hMG-treated group, the number density and total number of the primordial follicles decreased compared to the low-dose hMG-treated group (table 1).

*Hormonal Assay*

ND administration led to a significant decrease in the levels of gonadotropins and sex hormones in the blood.

As is summarized in table 2, there was a significant decrease in the levels of FSH (P=0.014)LH (P=0.014) estrogen (P=0), and progesterone (P≤0.001) in the low-dose ND-treated group and there was a significant decrease in the levels of FSH (P=0), LH (P=0), estrogen (P=0), and progesterone (P=0) in the high-dose ND-treated group.

**Table 2:** Mean value and standard deviation (SD) of FSH, LH, estrogen, and progesterone levels (ng/mL) in the sera of the rats treated with low and high doses of ND, alone or with hMG, and vehicle groups

	**FSH level (ng/mL) ** **mean±SD**	**LH level (ng/mL) ** **mean±SD**	**Estrogen (ng/mL) ** **mean±SD**	**Progesterone (ng/mL) ** **mean±SD**
LDN	2.0400±0.55299^a^	6.6960±0.43935^a^	24.9880±1.14262^b^	6.9380±0.66594^c^
HDN	1.8080±0.42222^d^	6.0960±0.52195^d^	23.3420±1.51206^d^	6.5640±0.96389^d^
Vehicle 1	2.8240±0.64891	7.5320±0.71963	26.0200±0.46583	8.4060±0.41010
LDN+hMG	1.9140±0.42477^e^	6.9020±0.44087^e^	26.5400±1.16319^e^	7.2260±0.38559^e^
HDN+hMG	2.1620±0.64005^f^	6.9200±0.41503^f^	25.7400±1.10589^f^	7.1280±1.01312^f^
Vehicle 2	2.9980±0.45185	7.7220±0.45240	27.4000±0.86023	8.1140±0.44925

a: Compared to vehicle1 (P=0.014); b: Compared to vehicle 1 (P=0); c: Compared to vehicle 1 (P≤0.001); d: Compared to vehicle 2 (P=0); e: Compared to vehicle 2 (P=0.037); f: Compared to vehicle 2 (P=0.022); hMG: Human menopausal gonadotropin; LDN: Low-dose Nandrolone; HDN: High-dose Nandrolone; Vehicle 1 (olive oil), vehicle 2 (olive oil+saline)

There was a significant decrease in the levels of FSH, LH, estrogen, and progesterone in the low-dose hMG-treated group (P=0.037) and the high-dose hMG-treated group (P=0.022). The hormone levels were not affected by hMG administration compared to the low- and high-dose ND-treated groups.

## Discussion

Ovarian follicles play an important role in the female reproductive biology. Gonadotropin-releasing hormone (GnRH) stimulates the release of FSH and LH from the anterior pituitary gland, and these hormones will later have a stimulatory effect on follicle growth. Research has shown that early follicular growth is regulated by a variety of stimulatory and inhibitory hormones and growth factors.^12^ AASs such as ND affect the structure and function of the reproductive system via impacting the secretion of FSH and LH through a negative feedback mechanism.^5^^,^^13^

The hormonal assay of the present study showed a rise in the level of androgens and a drop in the levels of FSH, LH, estrogen, and progesterone in the blood circulation as well as the total and cortical volumes of the ovary and the number of the ovarian primordial follicles of the ND-treated rats. This result is in agreement with those obtained by Attardi et al.^14^Gao et al.^15^ and Karbalay-Doust et al.^13^These authors showed that androgen reduces gonadotropin release via a negative feedback mechanism and decreases estrogen secretion in rats and mice. Synthetic steroids such as Estradiol Valerate suppress the serum levels of LH, FSH, and sex hormones and lead to a decrease in the number of the primordial follicles in rats.^16^^,^^17^ND may break up the function of the neuroendocrine axis, thus modifying the ovarian function and reducing the release of gonadotropins.^18^ Such a decline in the levels of estrogen and progesterone in the blood may induce structural changes such as a decrease in the number of the preantral and antral follicles in female rats and mice. Therefore, a reduction in the number of primordial follicles may be attributed to a reduction in the female sex hormones. A decrease in the number of the primordial follicles in the ovary may give rise to a reduction in the volume of the ovarian cortex and, subsequently, the total ovarian volume. The destruction of various follicles following treatment with ND in rats has been demonstrated by Gerez et al.^19^ Elsewhere, Shirwalkar et al.^16^ reported that the exposure of adult rats to Estradiol Valerate results in the destruction of folliculogenesis and increase in apoptosis in the granulosa cells of secondary and antral follicles. This may suggest that the decrease in the volume of the ovary and cortex after ND administration is due to the reduction in the number of ovarian follicles.

Yoshiko et al.^20^ reported that Ethinyl Estradiol influences the granulosa cells and oocytes in mice and thus brings about the degeneration of primordial follicles. It seems that the interaction between degenerative follicular cells and oocytes triggers the degeneration of primordial follicles.

The present study revealed that the administration of hMG with a low dose of ND (3 mg/kg) led to an increase in the total volume of the ovary, its cortical region, and the number of the primordial follicles compared with a high dose of ND (10 mg/kg) in rats. Our literature review showed that gonadotropins can be considered a primary factor for the survival and maintenance of ovarian follicles. Wang et al.^7^ demonstrated that hMG increases the survival of ovarian follicles by the induction of the expression of vascular endothelial growth factor (VEGF) and improves blood supply reconstruction in the ovarian tissue in mice. We also observed congestion in the ovarian medulla. (The data are not shown.) Congestion after ND administration in the other organs such as the liver has also been reported in rabbits. The toxic effects of ND on the liver may change the gonadotropin degradation by the liver.^21^ It seems that ND may also affect the metabolism of the hormones secreted by the hypothalamus and pituitary gland via interfering with the liver function in humans.

Warren,^22^ showed that exercise-induced amenorrhea occurs in women athletes. He demonstrated that these women appear to have pulsatility of FSH and LH due to environmental and metabolic stresses. Forceful exercise can restrain GnRH secretion, which is necessary for normal sexual and folliculogenesis progress in women. This situation occurs in highly competitive athletes. Camargo et al.^5^ showed the ND effects on the ovarian function and reduced sex hormones in rats. This may be the result of the hormonal dysfunction at the hypothalamic level and suppression of the normal pulsatile secretion of GnRH.

Gonadotropins also regulate the expression of P450 oxidoreductase and affect follicular development via steroidogenesis in rats. Gonadotropins can also be considered the primary survival factors for ovarian follicles.^23^ Ovarian growth factors may be responsible for the follicular survival mediated by gonadotropins. The findings of the present study showed that hMG may affect follicular development, survival, and maintenance by regulating ovarian growth factors via a direct action on the ovary. Folliculogenesis, induced by hMG administration, can lead to an increase in the number of ovarian follicles and, subsequently, the ovarian volume.

In conclusion, our experiments showed that ND reduced the volume of the ovary and the number of primordial follicles in low and high-dose ND-treated rats. Moreover, the administration of the gonadotropin, hMG, prevented the loss of the volume of the ovary and the number of the primordial follicles when the dose of ND was low.
